# Transforaminal versus interlaminar endoscopic approach for broad-based lumbar disc herniation under standardized postoperative mecobalamin therapy: a retrospective comparative study based on VAS and ODI

**DOI:** 10.3389/fneur.2026.1740615

**Published:** 2026-06-18

**Authors:** Haoran Gao, Yongxin Zhou, Kejun Hu, Lei Wang, Yu Chao

**Affiliations:** Department of Orthopedics, The First Affiliated Hospital of Xi’an Medical University, Xi'an, China

**Keywords:** broad-based lumbar disc herniation, interlaminar approach, mecobalamin, minimally invasive surgery, transforaminal approach

## Abstract

**Objective:**

This study aims to compare the clinical outcomes of the transforaminal approach (TFA) and interlaminar approach (ILA) for broad-based lumbar disc herniation (BLDH) under a standardized postoperative mecobalamin regimen. The primary focus is on dynamic changes in Visual Analog Scale (VAS) pain scores and Oswestry Disability Index (ODI) outcomes.

**Methods:**

This retrospective study included 184 patients with single-segment BLDH treated between June 2023 and October 2024 (89 in the TFA group and 95 in the ILA group). Both groups received the same postoperative mecobalamin regimen. Outcomes included the prespecified primary outcome of VAS score at 3 months postoperatively, as well as secondary outcomes including ODI, operative parameters, modified MacNab efficacy, neurological function, complications, and quality of life. Multivariable-adjusted analyses were performed to reduce potential selection bias.

**Results:**

Compared with TFA, ILA was associated with lower VAS scores at 3 months postoperatively (the prespecified primary outcome) and lower ODI at 3 days postoperatively (both *p* < 0.05). Additionally, the ILA group showed shorter operative time, fewer intraoperative fluoroscopies, and a greater volume of removed nucleus pulposus (all *p* < 0.05). Neurological indicators improved in both groups after treatment, without significant between-group differences. Clinical efficacy and complication rates were comparable between groups. In adjusted analyses, ILA remained significantly associated with lower ODI at 3 days (*B* = −2.883, 95% *CI*: −4.911 to −0.855, *p* = 0.006) and lower VAS scores at 3 months (*B* = −0.707, 95% *CI*: −0.930 to −0.485, *p* < 0.001).

**Conclusion:**

Under the same standardized postoperative mecobalamin treatment background, ILA is associated with better early postoperative recovery than TFA in patients with BLDH. These findings primarily reflect differences between the two surgical approaches rather than an independent effect of mecobalamin.

## 1 Introduction

Low back pain (LBP) affects approximately 60–80% of individuals at some point during their lifetime and represents one of the leading causes of disability worldwide (1). Lumbar disc herniation (LDH) accounts for nearly 5–10% of all LBP cases and is the most common cause of radicular leg pain, with an estimated annual incidence of 5–20 cases per 1,000 adults (2). Among patients with symptomatic LDH, broad-based lumbar disc herniation (BLDH) constitutes a substantial proportion and is characterized by disc protrusion involving more than 25% of the disc circumference, often resulting in extensive neural compression and persistent radiculopathy (3, 4). Nevertheless, the intricate anatomical complexity of BLDH, coupled with a high propensity for nerve root adhesions, often renders singular surgical approaches inadequate. Technical constraints such as limited operative visibility or incomplete decompression frequently culminate in suboptimal postoperative outcomes with persistent symptomatology (5). This clinical conundrum necessitates the development of optimized endoscopic strategies complemented by adjuvant therapies to enhance treatment efficacy. Mecobalamin, the physiologically active form of vitamin B12 (also known as methylcobalamin), is considered a novel adjuvant therapy for LDH (6). Its therapeutic mechanism, mediated through the promotion of nerve myelin regeneration and axonal repair, effectively mitigates nerve root damage (7). Although studies on transforaminal approach (TFA) or interlaminar approach (ILA) for the treatment of LDH are not uncommon, reports on their combination with mecobalamin are rare, with only a retrospective analysis conducted by Chen-Yang et al. (8). Notably, there exists a paucity of rigorous comparative analyses examining the clinical performance of different endoscopic approaches for the BLDH subtype under a similar postoperative treatment background. Furthermore, while the Visual Analog Scale (VAS) and Oswestry Disability Index (ODI) serve as gold-standard metrics for assessing LDH treatment outcomes, systematic investigations evaluating how surgical approach selection relates to these critical parameters in patients receiving the same postoperative adjunctive therapy remain limited.

Given these limitations, the present study compared two endoscopic techniques, TFA and ILA, in patients with BLDH who received the same standardized postoperative mecobalamin regimen. Through a comparative analysis of postoperative changes in VAS score and ODI, this study aimed to clarify the relative clinical performance of the two surgical approaches under complex anatomical conditions. This investigation may provide clinically relevant evidence for selecting surgical approaches in patients with BLDH. By incorporating multidimensional outcome assessments, the study seeks to improve the understanding of the comparative short- and medium-term outcomes of TFA and ILA under routine postoperative management.

## 2 Materials and methods

### 2.1 Study subjects

This retrospective study analyzed 184 BLDH patients who were admitted to our hospital between June 2023 and October 2024. The sample size was calculated based on the primary outcome (VAS score at 3 months postoperatively). According to preliminary clinical data and previous literature, a minimum clinically important difference (MCID) of 1.0 point on the VAS was assumed, with an estimated standard deviation of 2.0. Using a two-sided independent samples *t*-test, with a significance level (*α*) of 0.05 and statistical power (1–β) of 0.80, the required sample size was calculated to be 82 patients per group. Considering a potential 10% loss to follow-up, at least 90 patients per group were required. The sample size calculation was performed using G*Power software (version 3.1). All patients underwent preoperative MRI and CT examinations to evaluate disc morphology, confirm the involved segment, and assess the presence of calcified disc components. Calcified disc status was recorded as a baseline imaging characteristic.

Inclusion Criteria: (1) Meeting the diagnostic criteria for BLDH (9), confirmed by clinical symptoms, imaging examinations, and a positive straight leg raise test, with all cases representing first-time occurrences; (2) Single-segment BLDH localized to either L4/5 or L5/S1; (3) At least 3 months of standardized nonoperative management, including nonsteroidal anti-inflammatory drugs, neuropathic pain medications (e.g., pregabalin), structured physical therapy, and activity modification. Short-term corticosteroids or epidural steroid injections were used when indicated. Opioids were not routinely administered. Treatment failure was defined as persistent symptoms with a VAS score ≥ 4 despite conservative therapy; (4) Availability of complete clinical data.

Exclusion Criteria: (1) History of recurrent or multi-segment LDH; (2) Concurrent lumbar dynamic instability or lumbar spondylolisthesis; (3) Diagnosis of disc tuberculosis or spinal deformities; (4) Presence of malignant tumors; (5) Extensive lumbar spinal stenosis; (6) Severe comorbidities affecting major organs (heart, liver, kidney, or lung) that would contraindicate surgical intervention; (7) Far-lateral disc herniation; (8) Pregnancy or lactation; (9) Documented iodine allergy; (10) Severe psychiatric disorders, cognitive impairment, or consciousness disturbances.

The main clinical manifestations included low back pain, unilateral or bilateral radicular leg pain, lower limb numbness, and activity limitation. Most patients presented with unilateral radicular symptoms corresponding to the affected segment. Clinical symptom distribution was additionally recorded and compared between the two groups.

**Figure 1:**
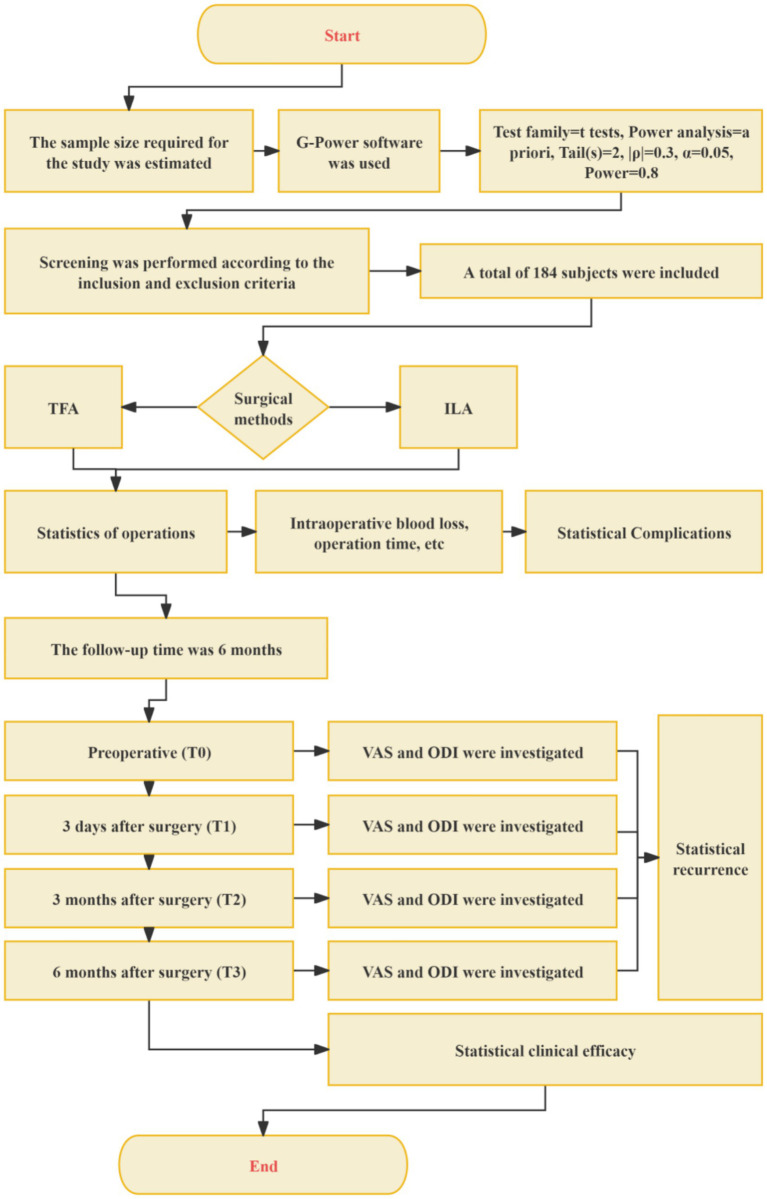
The main flow of this study.

Figure 1 illustrates the detailed screening process and study flowchart. The study protocol received approval from our hospital’s Ethics Committee and was conducted in full compliance with the principles outlined in the *Declaration of Helsinki*. Because this study involved a retrospective analysis of anonymized clinical data and posed minimal risk to participants, the requirement for informed consent was waived by the Ethics Committee.

The choice of surgical approach (TFA or ILA) was determined based on detailed preoperative imaging assessment, including the location of disc herniation, the degree of foraminal stenosis, and anatomical feasibility. Patients with far-lateral disc herniation were excluded. In cases of severe bony foraminal stenosis, the TFA was preferentially selected. The ILA was applied to patients in whom adequate decompression of the traversing and exiting nerve roots could be achieved via medial access under endoscopic visualization. All patients received standardized postoperative mecobalamin therapy according to the institutional protocol; therefore, the effect of the surgical approach was evaluated under the same adjuvant treatment background.

## 3 Methods

### 3.1 Surgical procedure

Patients in both groups underwent surgery performed by the same experienced surgical team, and pre- and post-operative nursing care was provided by the same nursing team at our hospital. All patients received epidural anesthesia, with the L2-L3 or L3-L4 intervertebral space selected (1–2 levels above the surgical segment to avoid affecting the surgical area). The skin and deep tissues at the puncture site were infiltrated with 1% lidocaine. A 17-18G Tuohy needle was inserted via a midline or paramedian approach. Then, 3 mL of 1.5% lidocaine with 1:200000 epinephrine was injected. After observing for 5 min, an additional 8–10 mL of 0.5–0.75% ropivacaine was administered, with the dose adjusted until the block plane reached T10–S1, covering the surgical segment.

TFA: The patient was positioned supine with the abdomen suspended and hips/knees flexed. Additionally, the surgical table was adjusted to minimize lumbar lordosis. The target vertebral segment was identified and marked under fluoroscopic guidance using a C-arm X-ray system. The puncture site was determined 12–14 cm lateral to the posterior midline. Using an 18-gauge puncture needle, access was established through the posterolateral Kambin’s triangle into the intervertebral foramen. A sequential dilation technique was then employed, introducing a guidewire followed by a dilating cannula and working channel. Subsequently, the spinal endoscope system was introduced, and under direct endoscopic visualization, periforaminal soft tissues were carefully dissected. Foraminoplasty was performed, which included partial resection of the superior articular process when required, to adequately expose the dural sac and decompress the affected nerve root. Thereafter, the annulus fibrosus was circumferentially incised, and herniated nucleus pulposus fragments were meticulously removed using nucleus forceps until complete nerve root decompression was achieved. Hemostasis was confirmed under endoscopic visualization before all instruments were systematically withdrawn.

ILA: The patient was maintained in the same surgical position as described above. A puncture point was marked 1.5 cm lateral to the midline of the interlaminar space. Following layered incision of the skin and fascia, a dilating cannula was advanced to the surface of the ligamentum flavum to establish a working channel. Under endoscopic visualization, partial resection of the ligamentum flavum was performed to widen the interlaminar space, thereby exposing the dural sac and nerve root. The nerve root was then carefully retracted to visualize the herniated nucleus pulposus. The posterior longitudinal ligament and annulus fibrosus on the symptomatic side were incised under endoscopic visualization, followed by complete removal of the herniated disc material.

Postoperative Management: Both patient groups remained on strict bed rest for the first 24 h following surgery. All participants wore protective lumbar braces for 4 weeks postoperatively while gradually initiating functional rehabilitation exercises for their lumbar back muscles. To facilitate nerve recovery, patients received daily intramuscular injections of 0.5 mg mecobalamin (Changchun Haiyue Pharmaceutical Co., Ltd., H20066982) for 14 consecutive days following the procedure.

### 3.2 Follow-up protocol

All enrolled patients completed a 6-month postoperative follow-up after discharge, with clinical assessments conducted through scheduled outpatient visits. Each participant was required to attend follow-up examinations at least once a month. At the last follow-up, patients’ quality of life was assessed using the ‌Medical Outcomes Study 36-Item Short Form Health Survey‌ (SF-36) (10). The SF-36 includes eight survey dimensions: Physical Functioning (PF), Role Physical (RP), Bodily Pain (BP), General Health (GH), Vitality (VT), Social Functioning (SF), Role Emotional (RE), Mental Health (MH). Higher scores indicate better quality of life. All 184 patients completed the 6-month follow-up. No patients were lost to follow-up due to the structured outpatient follow-up system and regular telephone reminders implemented by our institution.

### 3.3 Outcome measures

The prespecified primary outcome was the VAS score at 3 months postoperatively (T2), which was also used for sample size estimation. Secondary outcomes included ODI at different follow-up time points, operative parameters, intraoperative neurophysiological monitoring results, inflammatory and stress markers, neurological recovery indicators, modified MacNab efficacy, complications, recurrence, quality of life, and economic outcomes.

1. During the operation, the Medtronic NIM-Eclipse neuromonitor (model: 3.0) was used for continuous neurophysiological monitoring, including motor evoked potentials (MEP) [stimulating electrodes were placed in the motor cortex bilaterally over the tibialis anterior/gastrocnemius muscles to record compound muscle action potentials (CMAP)], somatosensory evoked potentials (SEP) [median nerve/posterior tibial nerve stimulation, cortical P40/N50 amplitude recording], and spontaneous electromyography (sEMG) [real-time monitoring of lower limb muscle activity to prevent intraoperative nerve root traction injury]. If MEP amplitude decreased by more than 50% or SEP latency prolonged by more than 10% during the operation, the procedure was immediately suspended, and the causes were investigated.
2. Surgical outcomes were recorded, including intraoperative blood loss, operative time, frequency of C-arm fluoroscopy, incision length, weight of the removed nucleus pulposus, time to postoperative ambulation, and length of hospital stay.
3. Clinical efficacy was assessed at the 6-month follow-up using the modified MacNab criteria (11), with outcomes categorized as follows: Excellent: Complete resolution of pain symptoms, negative straight leg raise test, and full return to normal activities. Good: Marked alleviation of low back and leg pain, negative straight leg raise test, with the ability to resume normal work and daily activities. Fair: Partial relief of low back and leg pain, necessitating modifications to work or lifestyle. Poor: No improvement or exacerbation of low back and leg pain. The total effective rate was defined as the sum of the excellent and good rates.
4. Pain intensity and lumbar functional recovery were evaluated using the VAS (12) and ODI (13) preoperatively (T0), 3 days postoperatively (T1), 3 months postoperatively (T2), and 6 months postoperatively (T3). The VAS score ranges from 0 (no pain) to 10 (unbearable pain), while the ODI comprises 10 items totaling 100 points, with higher scores indicating worse lumbar function.
5. Fasting venous blood samples were collected before surgery and 3 days after surgery. Inflammatory and stress response markers were detected by enzyme-linked immunosorbent assay (ELISA) to compare the postoperative inflammatory and stress response between the two groups. These cytokines included interleukin-1β/6 (IL-1β/6), tumor necrosis factor-*α* (TNF-α), superoxide dismutase (SOD) and malondialdehyde (MDA).
6. Fasting venous blood samples were collected from patients before and after treatment (3 months after surgery), and the levels of nerve growth factor (NGF) and brain-derived neurotrophic factor (BDNF) were measured using ELISA. In addition, electromyography (EMG) was used to assess nerve conduction velocity (NCV) and CMAP.
7. Postoperative complications (e.g., nerve root injury, dural tear) during hospitalization and recurrence of BLDH were documented.
8. An exploratory economic comparison was performed based on direct in-hospital medical costs, including operation cost, anesthesia cost, hospitalization cost, and drug cost. Total cost and cost per effective case were calculated for descriptive comparison between groups, with the total effective rate based on the modified MacNab criteria used as the clinical effectiveness measure.

### 3.4 Statistical analysis

To mitigate potential selection bias inherent in retrospective studies, strict inclusion and exclusion criteria were applied, and baseline characteristics between groups were carefully compared to ensure comparability. All surgical procedures were performed by the same experienced surgical team, and postoperative management was standardized to reduce performance bias. Outcome assessments were conducted according to predefined protocols, and objective measurement tools (VAS, ODI, neuroelectrophysiological monitoring, and laboratory indices) were used to minimize measurement bias.

All statistical analyses were performed using SPSS 25.0. Categorical variables are presented as frequencies and percentages [n (%)], and group comparisons were assessed using the chi-square test. The normality of continuous variables was evaluated using the Shapiro–Wilk test. Normally distributed data are expressed as ( $\bar{x}$ ± s), with between-group differences analyzed by independent samples *t*-tests and within-group comparisons assessed using paired *t*-tests. For non-normally distributed data, results are presented as median [interquartile range (IQR)], with between-group comparisons performed using the Mann–Whitney *U* test and within-group comparisons analyzed by the Wilcoxon signed-rank test. For longitudinal VAS and ODI outcomes measured at multiple time points (T0, T1, T2, and T3), linear mixed-effects models were additionally applied to evaluate the effects of group, time, and the group-by-time interaction. A random intercept was included to account for within-subject correlations. When the overall model was significant, *post hoc* pairwise comparisons with Bonferroni correction were performed. Effect estimates with 95% confidence intervals (*CI*s) were reported. To reduce potential selection bias associated with the retrospective and non-randomized design, additional multivariable-adjusted analyses were performed for the main outcomes. A *p*-value < 0.05 was considered statistically significant.

## 4 Results

### 4.1 The two groups demonstrate no significant differences in baseline characteristics

No significant differences were observed in baseline clinical and imaging characteristics between the two groups, including age, sex, disease duration, affected segment, symptom laterality, Pfirrmann classification, and calcified disc status (all *p* > 0.05), confirming group comparability (Table 1).

**Table 1:** Comparison of baseline data.

Groups	n	Age (years)	Male vs. female	Duration since diagnosis (months)	Diseased segments L4/5 vs. L5_/_S1	BMI (kg/m^2^)	Pfirrmann classification II vs. III vs. IV	Calcified disc (%)	Unilateral symptoms, n (%)	Bilateral symptoms, n (%)
									Unilateral symptoms, n (%)	Bilateral symptoms, n (%)
TFA	89	55.03 ± 7.64	54 (60.67%) *vs.* 35 (39.33%)	22.93 ± 6.37	47 (52.81%) *vs.* 42 (47.19%)	24.73 ± 2.68	42 (47.19%) *vs.* 33 (37.08%) *vs.* 14 (15.73%)	21 (23.6%)	76 (85.39%)	13 (14.61%)
ILA	95	56.55 ± 7.92	62 (65.26%) *vs.* 33 (34.74%)	21.52 ± 7.55	55 (57.89%) *vs.* 40 (42.11%)	24.80 ± 2.61	39 (41.05%) *vs.* 43 (45.26%) *vs.* 13 (13.68%)	24 (25.3%)	83 (87.37%)	12 (12.63%)
*t*/*χ^2^*		1.318	0.415	1.370	0.481	0.178	1.270	0.072	0.151	
*P*		0.189	0.519	0.172	0.488	0.859	0.530	0.789	0.698	

TFA, transforaminal approach; ILA, interlaminar approach; BMI, body mass index.

### 4.2 Primary outcome and longitudinal VAS/ODI outcomes

**Figure 2:**
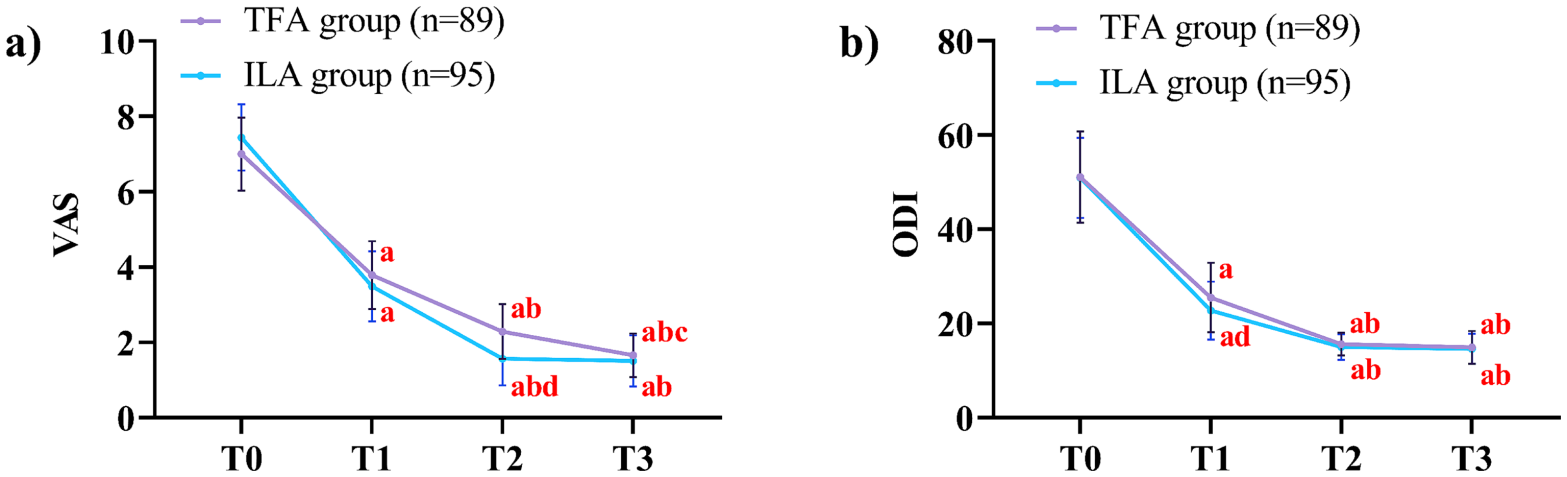
Longitudinal changes in VAS and ODI scores across follow-up time points in the TFA and ILA groups. Repeated-measures analysis was performed using linear mixed-effects models with Bonferroni-corrected *post hoc* comparisons. **(a)** Comparison of VAS between the two groups. **(b)** Comparison of ODI between the two groups. a denotes *p* < 0.05 compared with T0, b denotes *p* < 0.05 compared with T1, c denotes *p* < 0.05 compared with T2, and d denotes *p* < 0.05 compared with the TFA group. VAS, Visual Analog Scale; ODI, Oswestry Disability Index; TFA, transforaminal approach; ILA, interlaminar approach.

Longitudinal analysis using linear mixed-effects models demonstrated significant overall effects of time on both VAS and ODI scores, indicating progressive postoperative improvement in both groups (both *p* < 0.001; Table 2). For VAS, significant effects of group and group-by-time interaction were also observed (both *p* < 0.001), suggesting that postoperative pain trajectories differed between the TFA and ILA groups over time. For ODI, the group effect and group-by-time interaction were not statistically significant (*p* = 0.844 and *p* = 0.140, respectively). *Post hoc* Bonferroni-corrected comparisons showed that the ILA group had significantly lower ODI scores at 3 days postoperatively (T1) and lower VAS scores at 3 months postoperatively (T2), whereas no significant between-group differences were observed at baseline (T0) or at 6 months (T3) (Figure 2). Estimated marginal mean differences further showed that the ILA group had lower VAS scores at T2 (*β* = −0.724, 95% *CI*: −0.930 to −0.517, *p* < 0.001) and lower ODI scores at T1 (*β* = −2.760, 95% *CI*: −4.722 to −0.797, *p* = 0.006) than the TFA group (Table 2).

**Table 2:** Linear mixed-effects analysis of longitudinal VAS and ODI outcomes.

Outcome	Effect	Wald *χ^2^*	Estimate (*β*)	95% *CI*	*p* value
VAS	Group	13.752	—	—	< 0.001
VAS	Time	3381.895	—	—	< 0.001
VAS	Group × Time	48.94	—	—	< 0.001
ODI	Group	0.039	—	—	0.844
ODI	Time	2281.482	—	—	< 0.001
ODI	Group × Time	5.481	—	—	0.140
VAS (T2)	ILA − TFA	—	−0.724	−0.930 to −0.517	< 0.001
ODI (T1)	ILA − TFA	—	−2.760	−4.722 to −0.797	0.006

Linear mixed-effects models were used to evaluate repeated measurements across T0, T1, T2, and T3, including the fixed effects of group, time, and the group-by-time interaction. VAS, Visual Analog Scale; ODI, Oswestry Disability Index; TFA, transforaminal approach; ILA, interlaminar approach; CI, confidence interval.

### 4.3 ILA contributes to better surgical outcomes

While intraoperative blood loss, incision length, time to postoperative ambulation, and hospital stay were comparable between the groups (*p* > 0.05), the ILA group demonstrated advantages in operative time (shorter), frequency of C-arm fluoroscopy (fewer), and weight of the removed nucleus pulposus (greater) compared to the TFA group (*p* < 0.05) (Table 3).

**Table 3:** Comparison of surgical situations.

Groups	*n*	Operative time (min)	Intraoperative blood loss (mL)	Frequency of C-arm fluoroscopy	Weight of nucleus pulposus removed (g)	Incision length (mm)	Time to postoperative ambulation (h)	Length of hospital stay (d)
TFA	89	85.51 ± 8.50	26.61 ± 4.38	5.63 ± 2.60	2.71 ± 0.59	7.67 ± 0.45	22.04 ± 3.93	3.97 ± 1.03
ILA	95	75.75 ± 8.48	25.47 ± 6.16	3.47 ± 1.49	3.44 ± 0.63	7.54 ± 0.60	21.63 ± 4.78	3.87 ± 1.18
*t*		7.793	1.430	6.959	8.100	1.583	0.638	0.567
*P*		< 0.001	0.154	< 0.001	< 0.001	0.115	0.524	0.572

TFA, transforaminal approach; ILA, interlaminar approach.

### 4.4 ILA has a lesser effect on neurological function

Compared with the TFA group, the changes in MEP amplitude and SEP latency in the ILA group were smaller (both *p* < 0.05), while the abnormal discharge rate on sEMG was higher (*p* = 0.033) (Table 4).

**Table 4:** Results of intraoperative neuroelectrophysiological monitoring.

Groups	n	MEP amplitude change (%)	SEP latency change (ms)	sEMG abnormal discharge rate (%)
TFA	89	7.81 ± 3.14	2.33 ± 1.07	13 (14.61)
ILA	95	6.50 ± 2.57	1.93 ± 0.97	5 (5.26)
*t*/*χ^2^*		3.195	2.649	4.545
*P*		0.002	0.009	0.033

TFA, transforaminal approach; ILA, interlaminar approach; MEP, motor evoked potential; SEP, somatosensory evoked potential; sEMG, spontaneous electromyography.

### 4.5 The two groups are similar in clinical efficacy

The total effective rate was similar between the ILA [91.58% (87/95)] and TFA [88.76% (79/89)] groups, with no statistically significant difference (*p* > 0.05) (Table 5).

**Table 5:** Comparison of clinical efficacy.

Groups	n	Excellent	Good	Fair	Poor	Total effective rate
TFA	89	43 (48.31%)	36 (40.45%)	5 (5.62%)	5 (5.62%)	79 (88.76%)
ILA	95	49 (51.58%)	38 (40.00%)	5 (5.26%)	3 (3.16%)	87 (91.58%)
*χ^2^*						0.413
*P*						0.521

TFA, transforaminal approach; ILA, interlaminar approach.

### 4.6 The inflammatory and stress response is less after ILA

**Figure 3:**
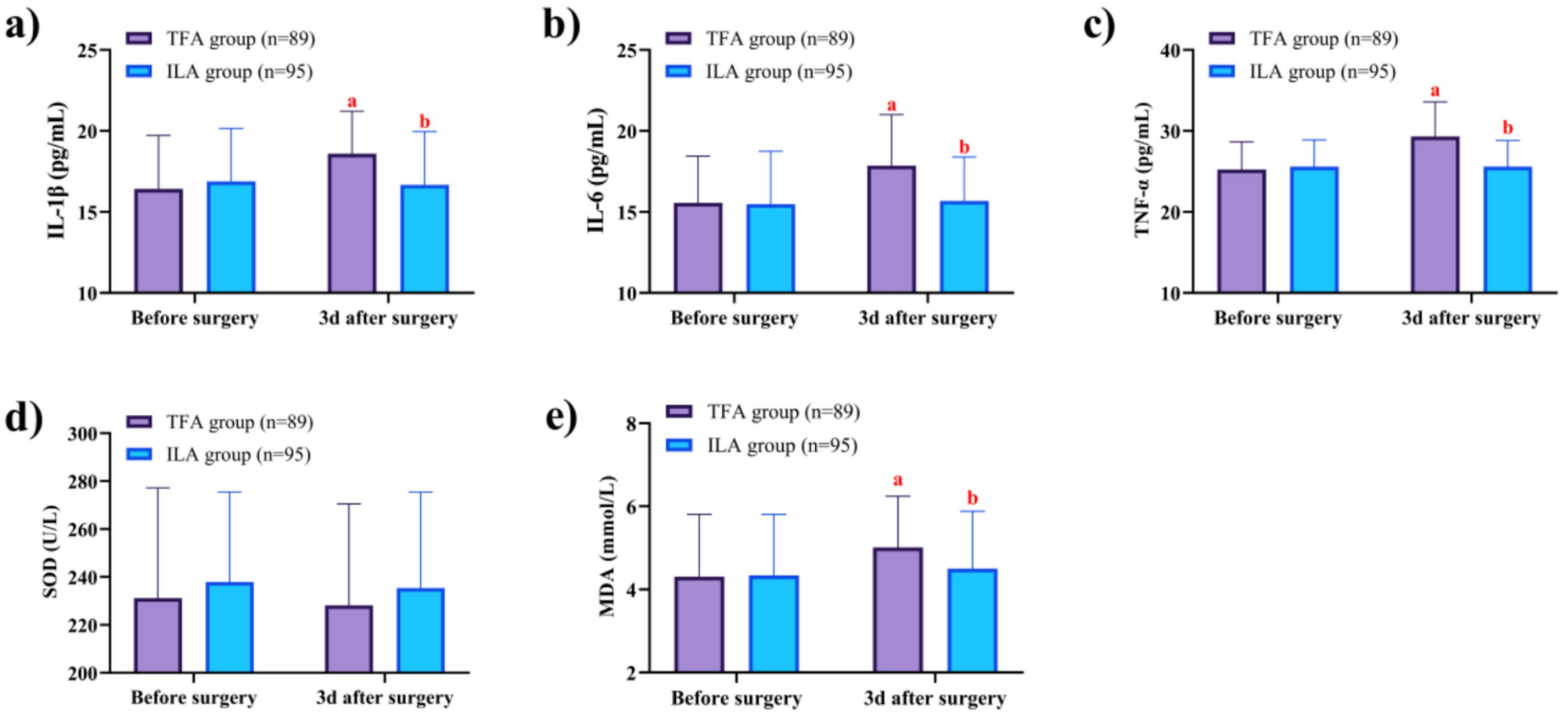
Comparison of inflammatory factors and markers of stress response. **(a)** Comparison of IL-1β between the two groups. **(b)** Comparison of IL-6 between the two groups. **(c)** Comparison of TNF-*α* between the two groups. **(d)** Comparison of SOD between the two groups. **(e)** Comparison of MDA between the two groups. a denotes *p* < 0.05 compared with before treatment. IL-1β, interleukin-1β; IL-6, interleukin-6; TNF-α, tumor necrosis factor-α; SOD, superoxide dismutase; MDA, malondialdehyde.

Before surgery, there were no significant differences in IL-1β, IL-6, TNF-*α*, SOD, or MDA levels between the two groups (*p* > 0.05). After surgery, inflammatory markers (IL-1β, IL-6, TNF-α) and MDA levels increased in both groups compared with preoperative levels. However, these markers were significantly higher in the TFA group than in the ILA group (*p* < 0.05), suggesting that the inflammatory and stress response was relatively milder following the ILA procedure (Figure 3).

### 4.7 No difference in neurological function between the two groups

**Figure 4:**
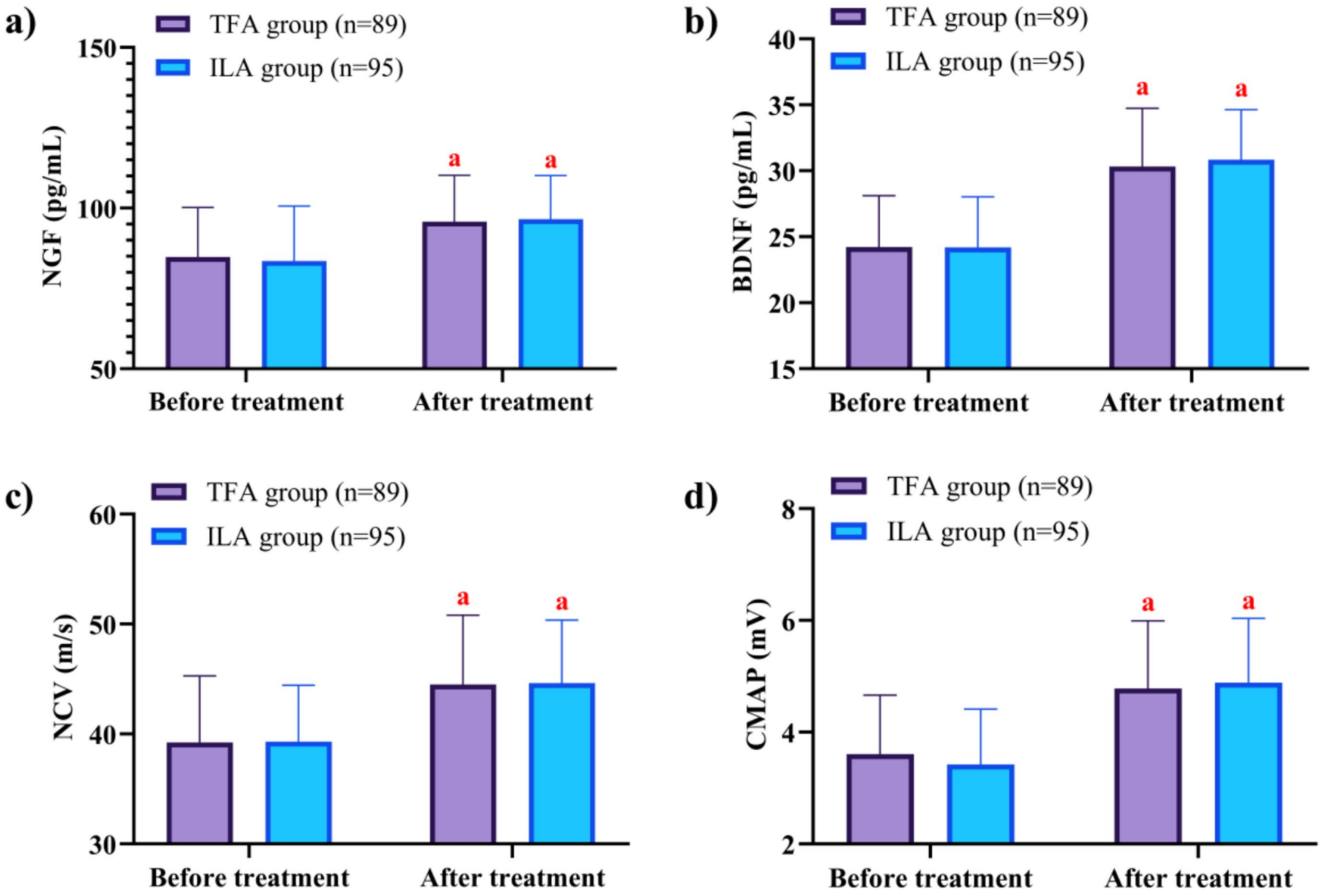
Comparison of neurological function. **(a)** Comparison of NGF between the two groups. **(b)** Comparison of BDNF between the two groups. **(c)** Comparison of NCV between the two groups. **(d)** Comparison of CMAP between the two groups. a denotes *p* < 0.05 compared with before treatment. NGF, nerve growth factor; BDNF, brain-derived neurotrophic factor; NCV, nerve conduction velocity; CMAP, compound muscle action potential.

A comparison of neurological function between the two groups showed no differences in NGF, BDNF, NCV, or CMAP before treatment (all *p* > 0.05). After treatment, NGF, BDNF, NCV, and CMAP increased in both groups compared with before treatment (all *p* < 0.05), but there were still no differences between the two groups (all *p* > 0.05) (Figure 4).

### 4.8 No significant inter-group differences in complication or recurrence rates are observed

In terms of safety, the incidence of complications did not differ significantly between the groups (*p* > 0.05). During follow-up, recurrence occurred in 3 patients in the ILA group and 5 in the TFA group, with no significant difference in the 6-month recurrence rate (*p* > 0.05). At the same time, the SF-36 results showed that the ILA group had higher scores in the SF and RE domains than the TFA group (both *p* < 0.05), suggesting that the ILA group returned to social participation earlier (Table 6).

**Table 6:** Comparison of safety and prognostic recurrence.

Variable		TFA (*n* = 89)	ILA (*n* = 95)	*χ^2^*/Fisher’s exact/*t*	*P*
Adverse reactions	Nerve root injury	3 (3.37%)	2 (2.11%)		
	Dural sac injury	2 (2.25%)	3 (3.16%)		
	Postoperative paresthesia	2 (2.25%)	3 (3.16%)		
	Residual nucleus pulposus	3 (3.37%)	1 (1.05%)		
	Total	10 (11.24%)	9 (9.47%)	0.154	0.695
Recurrence rate		5 (5.62%)	3 (3.16%)	-	0.49
SF-36	PF	72.56 ± 5.62	74.06 ± 5.82	1.778	0.077
	RP	74.16 ± 5.43	74.49 ± 5.63	0.413	0.680
	RE	73.34 ± 5.43	76.52 ± 6.89	2.372	0.019
	BP	73.26 ± 5.50	74.29 ± 5.51	1.276	0.204
	GH	73.62 ± 6.16	73.46 ± 5.69	0.177	0.860
	SF	72.98 ± 5.62	74.96 ± 6.72	2.161	0.032
	MH	74.76 ± 5.51	74.71 ± 5.12	0.075	0.940
	VT	73.91 ± 5.49	74.23 ± 5.73	0.388	0.699

TFA, transforaminal approach; ILA, interlaminar approach; PF, Physical Functioning; RP, Role Physical; RE, Role Emotional; BP, Bodily Pain; GH, General Health; SF, Social Functioning; MH, Mental Health; VT, Vitality.

### 4.9 Comparison of in-hospital costs and cost per effective case

No significant difference was observed in total direct medical costs between the two groups (*p* > 0.05). A descriptive comparison of the cost-effectiveness ratio (CER) based on the total effective rate was additionally performed (Table 7). Given the limited scope of the available economic data and the absence of a formal health-economic framework, these findings should be interpreted as exploratory only.

**Table 7:** Comparison of in-hospital costs and cost per effective case.

Groups	n	Total cost (Yuan)	CER
TFA	89	23503.73 ± 1683.05	297.52 ± 21.30
ILA	95	23992.33 ± 2086.14	275.77 ± 23.98
*t*		1.741	6.485
*P*		0.083	< 0.001

TFA, transforaminal approach; ILA, interlaminar approach; CER, cost-effectiveness ratio.

### 4.10 Representative cases

**Figure 5:**
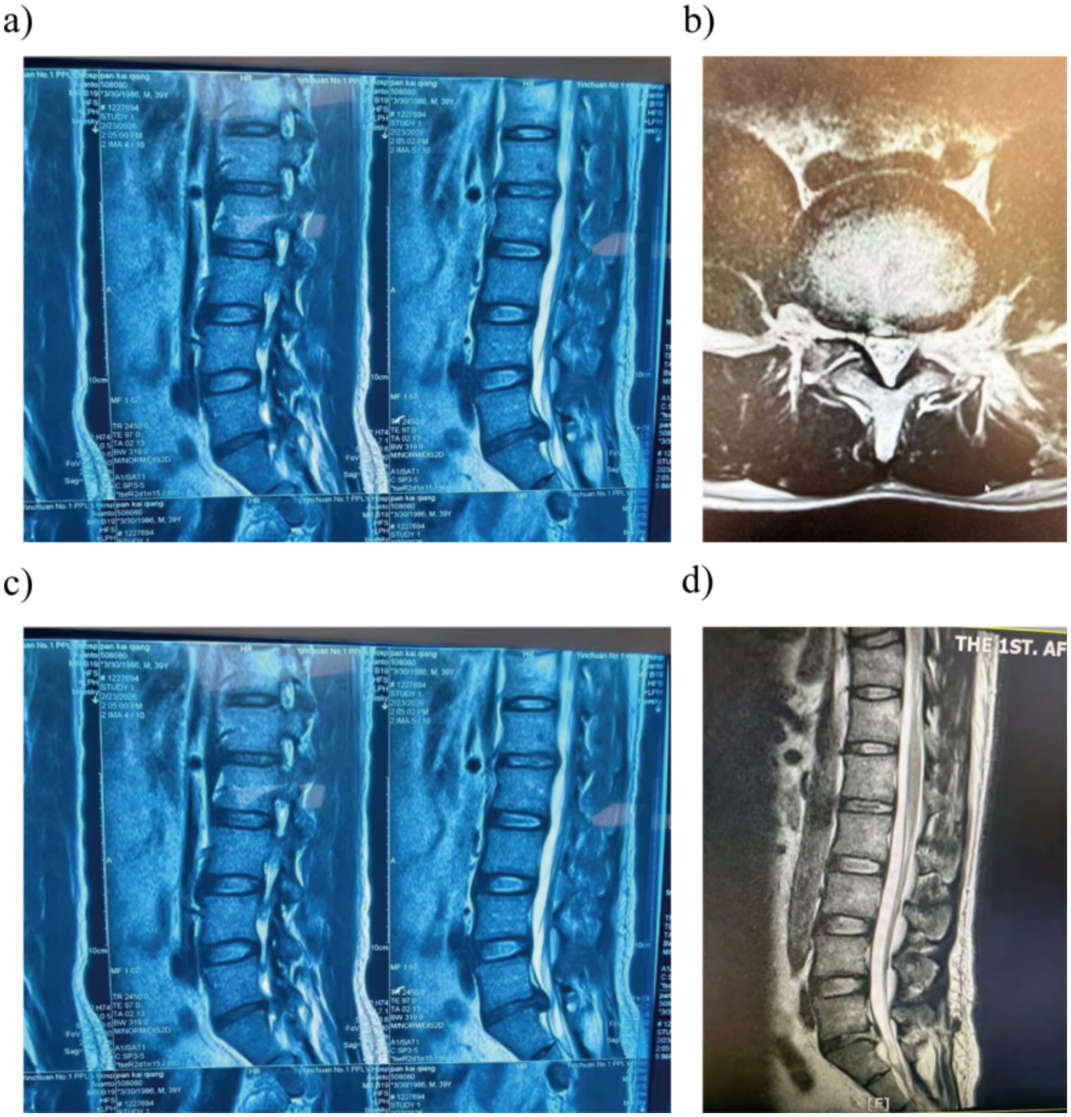
Representative MRI images. **(a)** TFA preoperative MRI. **(b)** TFA postoperative MRI. **(c)** ILA preoperative MRI. **(d)** ILA postoperative MRI. TFA, transforaminal approach; ILA, interlaminar approach; MRI, magnetic resonance imaging.

**Figure 6:**
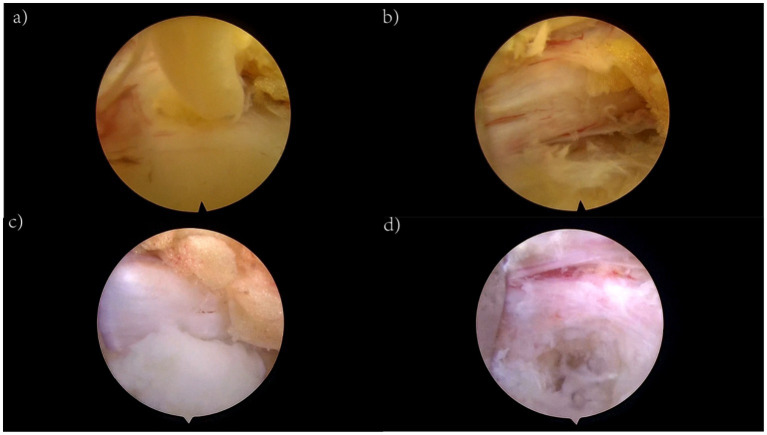
Representative intraoperative endoscopic views during neural decompression in the TFA and ILA groups. **(a)** TFA intraoperative visualization during nucleus pulposus removal. **(b)** TFA after decompression. **(c)** ILA intraoperative visualization of the herniated disc. **(d)** ILA after decompression following disc removal. TFA, transforaminal approach; ILA, interlaminar approach.

Representative preoperative and postoperative MRI images from one patient in each group are shown in Figure 5. The images illustrate the typical radiological characteristics of BLDH and the postoperative decompression achieved by the two surgical approaches. Representative intraoperative endoscopic views during neural decompression are presented in Figure 6, including representative views obtained during nucleus pulposus removal and after decompression. These images further demonstrate the different surgical visualization fields and decompression characteristics between the TFA and ILA approaches under endoscopic guidance.

### 4.11 Adjusted analysis of the primary and main secondary outcomes

To further reduce potential selection bias related to the retrospective and non-randomized design, multivariable-adjusted analyses were performed for the main outcomes (Tables 8A–D). After adjustment for age, sex, BMI, disease duration, affected segment, Pfirrmann classification, and baseline VAS/ODI where appropriate, the ILA group remained significantly associated with lower ODI scores at 3 days postoperatively (*B* = −2.883, 95% *CI*: −4.911 to −0.855, *p* = 0.006) and lower VAS scores at 3 months postoperatively (*B* = −0.707, 95% *CI*: −0.930 to −0.485, *p* < 0.001). However, no significant associations were observed between surgical approach and total clinical efficacy (*OR* = 1.475, 95% *CI*: 0.534–4.076, *p* = 0.453) or postoperative complications (*OR* = 0.650, 95% *CI*: 0.212–1.995, *p* = 0.451). Overall, the adjusted results were consistent with the primary unadjusted analyses.

**Table 8:** Multivariable-adjusted analyses of the main outcomes

Variable	*B*	*SE*	*t*	95% *CI*		*P* value
A. Adjusted linear regression for ODI at T1						
Intercept	32.643	6.414	5.089	19.984 to 45.303		< 0.001
Pfirrmann grade III (*vs.* II)	0.507	1.109	0.457	−1.683 to 2.696		0.648
Pfirrmann grade IV (*vs.* II)	−0.71	1.55	−0.458	−3.769 to 2.350		0.648
ILA (*vs.* TFA)	−2.883	1.027	−2.806	−4.911 to −0.855		0.006
Age, years	0.024	0.067	0.35	−0.109 to 0.156		0.727
Male (*vs.* female)	−0.014	1.077	−0.013	−2.139 to 2.112		0.99
BMI, kg/m^2^	−0.303	0.198	−1.532	−0.693 to 0.087		0.127
Disease duration, months	0.009	0.074	0.123	−0.137 to 0.155		0.902
L5/S1 (*vs.* L4/5)	−0.772	1.045	−0.738	−2.835 to 1.291		0.461
Baseline ODI	−0.016	0.058	−0.283	−0.130 to 0.097		0.777
B. Adjusted linear regression for VAS at T2						
Intercept	2.359	0.798	2.957		0.784 to 3.933	0.004
Pfirrmann grade III (*vs.* II)	−0.038	0.118	−0.32		−0.271 to 0.195	0.749
Pfirrmann grade IV (*vs.* II)	−0.056	0.165	−0.339		−0.381 to 0.269	0.735
ILA (*vs.* TFA)	−0.707	0.113	−6.266		−0.930 to −0.485	< 0.001
Age, years	−0.003	0.007	−0.401		−0.017 to 0.011	0.689
Male (*vs.* female)	−0.079	0.114	−0.69		−0.304 to 0.147	0.491
BMI, kg/m^2^	0.021	0.021	1.002		−0.020 to 0.062	0.318
Disease duration, months	−0.004	0.008	−0.562		−0.020 to 0.011	0.575
L5/S1 (*vs.* L4/5)	−0.05	0.111	−0.448		−0.269 to 0.170	0.655
Baseline VAS	−0.033	0.06	−0.558		−0.152 to 0.085	0.577
C. Adjusted logistic regression for total clinical efficacy						
Intercept	1.574	3.025	0.271	4.828	0.013 to 1814.835	0.603
Pfirrmann grade III (*vs.* II)	−0.777	0.597	1.693	0.46	0.143 to 1.481	0.193
Pfirrmann grade IV (*vs.* II)	−1.291	0.747	2.984	0.275	0.064 to 1.190	0.084
ILA (*vs.* TFA)	0.389	0.519	0.562	1.475	0.534 to 4.076	0.453
Age, years	−0.007	0.033	0.052	0.993	0.931 to 1.058	0.819
Male (*vs.* female)	−0.19	0.545	0.122	0.827	0.284 to 2.405	0.727
BMI, kg/m^2^	0.073	0.099	0.545	1.076	0.886 to 1.306	0.461
Disease duration, months	−0.025	0.036	0.493	0.975	0.908 to 1.047	0.483
L5/S1 (*vs.* L4/5)	0.99	0.576	2.955	2.691	0.870 to 8.323	0.086
D. Adjusted logistic regression for postoperative complications						
Intercept	−2.093	3.402	0.378	0.123	0.000 to 96.952	0.538
Pfirrmann grade III (*vs.* II)	0.262	0.592	0.197	1.3	0.408 to 4.145	0.658
Pfirrmann grade IV (*vs.* II)	−0.726	1.117	0.422	0.484	0.054 to 4.322	0.516
ILA (*vs.* TFA)	−0.431	0.572	0.568	0.65	0.212 to 1.995	0.451
Age, years	0.007	0.038	0.034	1.007	0.935 to 1.085	0.853
Male (*vs.* female)	−0.009	0.593	0.000	0.992	0.310 to 3.171	0.989
BMI, kg/m^2^	−0.025	0.111	0.053	0.975	0.785 to 1.211	0.818
Disease duration, months	0.001	0.042	0.000	1.001	0.922 to 1.086	0.972
L5/S1 (vs L4/5)	−0.034	0.573	0.003	0.967	0.315 to 2.970	0.953

Model statistics: N = 184; R^2^ = 0.064; adjusted R^2^ = 0.016; F = 1.325; *p* = 0.227.

Model statistics: N = 184; R^2^ = 0.216; adjusted R^2^ = 0.176; F = 5.330; p < 0.001.

Model statistics: N = 184; pseudo R^2^ = 0.061; LR χ^2^ = 7.161; *p* = 0.519; AIC = 128.702.

Model statistics: N = 184; pseudo R^2^ = 0.016; LR χ^2^ = 1.620; *p* = 0.991; AIC = 115.412.

Covariates included in the adjusted models: age, sex, BMI, disease duration, affected segment, Pfirrmann classification, and baseline VAS/ODI where appropriate. Abbreviations: ODI, Oswestry Disability Index; VAS, Visual Analog Scale; ILA, interlaminar approach; TFA, transforaminal approach; BMI, body mass index; OR, odds ratio; CI, confidence interval; SE, standard error; LR, likelihood ratio; AIC, akaike information criterion.

## 5 Discussion

Due to the significant disc herniation and severe nerve compression associated with BLDH, this condition often leads to intractable radicular symptoms and functional impairments, posing significant challenges for clinical management. While traditional open surgery can effectively achieve nerve decompression, its invasiveness and prolonged recovery period limit its applicability. Although mecobalamin is commonly used as postoperative adjunctive therapy, few studies have systematically compared the clinical performance of TFA and ILA for BLDH under the same postoperative treatment background. In the present study, both groups received the same standardized postoperative mecobalamin regimen; therefore, our analysis primarily focused on the comparative technical and clinical outcomes of the two surgical approaches rather than the effect of mecobalamin itself. The introduction of intraoperative neuroelectrophysiological monitoring revealed differences in neuroprotection between the two procedures: MEP amplitude decreased less in the ILA group, which may be related to direct decompression reducing nerve traction. Representative intraoperative endoscopic images further illustrated the different visualization fields and decompression characteristics between the two approaches. This finding supports the advantage of ILA application in high-risk neuroanatomical structures.

First, regarding surgical outcomes, the ILA group demonstrated significantly shorter operative time, likely due to the direct exposure of the dural sac and nerve roots facilitated by ILA. By enlarging the interlaminar space, ILA provides clear visualization and efficient management of central disc herniation, offering a broader surgical field, particularly advantageous in cases of severe foraminal stenosis or calcified disc fragments (14). In contrast, TFA involves puncture through the Kambin’s triangle of the intervertebral foramen, often requiring foraminoplasty (e.g., partial resection of the superior articular process). This additional step increases procedural complexity, prolonging both fluoroscopy exposure and overall operative time (15). Moreover, the ILA group exhibited a greater volume of removed nucleus pulposus, indicating superior efficacy in addressing extensive disc herniation, a finding consistent with previous reports by Yin et al. (16).

In terms of prognostic rehabilitation, the ILA group demonstrated significantly better ODI scores at 3 days postoperatively (T1) and VAS scores at 3 months (T2) compared to the TFA group, indicating superior short-term recovery outcomes. This advantage may be attributed to the reduced nerve root traction associated with the ILA technique. As established in the literature, TFA necessitates access through the narrow intervertebral foramen. While this approach preserves the lamina, the puncture trajectory may increase mechanical irritation of the nerve roots, exacerbating transient postoperative inflammatory responses (17). In contrast, ILA involves direct removal of the herniated nucleus pulposus by gently retracting the nerve root. Although ligamentum flavum resection is required, the resulting surgical field offers enhanced visualization, facilitating more thorough decompression and consequently greater and earlier pain relief (18). Of particular note, by the 6-month follow-up (T3), the inter-group differences in VAS and ODI scores had resolved, suggesting that medium-term outcomes may be influenced by postoperative nerve recovery and the natural course after adequate decompression under standardized postoperative management, rather than a differential effect of adjunctive pharmacological therapy. The absence of significant inter-group differences in postoperative neurological function suggests that both TFA and ILA achieve comparable long-term nerve repair outcomes under the same standardized postoperative treatment background. Previous studies have demonstrated that mecobalamin may promote axonal regeneration and myelin sheath repair (19) and may also exert anti-inflammatory effects by modulating cytokines such as IL-6 and TNF-*α* (20). These findings provide a theoretical basis for its routine use as adjunctive therapy. However, because all patients in the present study received the same standardized postoperative mecobalamin regimen, our study was not designed to evaluate the independent therapeutic effect of mecobalamin or to determine whether any synergistic interaction exists between pharmacological treatment and surgical approach. Therefore, the observed improvements in VAS and ODI in both groups should primarily be interpreted as the result of surgical decompression under a uniform postoperative treatment background, rather than the effect of mecobalamin itself.

Similarly, although previous studies have suggested that methylcobalamin may have anti-inflammatory and neuroprotective properties (21), the between-group differences in inflammatory and stress responses observed in this study should still be interpreted mainly in relation to differences in surgical technique, since postoperative mecobalamin treatment was identical in both groups.

Furthermore, no significant differences were observed in clinical efficacy or recurrence rates between the two groups, indicating comparable overall outcomes for both surgical approaches under the same adjunctive treatment background. Similarly, we detected changes in inflammatory factors and stress responses before and after surgery in the two groups; these also showed that the inflammatory response in the ILA group was milder in the early postoperative period, which again verifies that ILA has the advantages of less surgical trauma and limited soft tissue dissection.

The results of the prognostic quality of life survey showed that the improvement in living ability in the ILA group was also significantly better than that in the TFA group, which is also attributable to the above-mentioned positive effects of ILA on patients. Although VAS and ODI can reflect pain and functional status, the social function dimension of the SF-36 reveals differences in the ability of patients to return to work, family, and society after surgery. This suggests that clinical decisions need to consider both biomedical indicators and psychosocial outcomes. No significant difference was observed in total direct medical costs between the two groups. An additional CER-based descriptive comparison was performed; however, because the present study did not adopt a formal health-economic framework and did not include broader cost components such as indirect costs or post-discharge expenditures, the economic findings should be interpreted cautiously and regarded as exploratory.

Based on the findings of this study, we propose the following clinical translation recommendations: First, surgical approaches should be carefully selected based on individual patient anatomy. TFA should be prioritized for cases with a high iliac crest obstruction (such as at L5/S1) or severe foraminal stenosis to avoid anatomical interference. Conversely, ILA may be more appropriate for central disc herniation or severe calcification to ensure complete decompression. Furthermore, ILA may be the preferred choice when rapid functional recovery is clinically important, as this approach is associated with shorter rehabilitation periods. Second, surgeons should receive specialized training to overcome the technical challenges associated with the TFA procedure. Targeted training programs should focus on reducing fluoroscopy frequency and operative time. Third, the development of more sophisticated endoscopic instruments could potentially improve the decompression efficiency of both TFA and ILA techniques.

Despite these findings, several limitations should be acknowledged. First, this was a retrospective and non-randomized study, and the choice of surgical approach was determined by preoperative imaging findings and anatomical feasibility, which may have introduced selection bias and confounding by indication. Although multivariable-adjusted analyses were performed to reduce this bias, residual confounding from unmeasured factors cannot be fully excluded. Second, because both groups received the same postoperative mecobalamin regimen, the present study could not assess the independent efficacy of mecobalamin or determine whether any synergistic effect existed between pharmacological treatment and surgical approach. Third, the follow-up duration of 6 months may not fully capture long-term recurrence and functional outcomes. Fourth, although standardized conservative treatment and surgical protocols were implemented, residual variability in clinical decision-making may still exist. Future multicenter randomized controlled trials with longer follow-up are warranted to validate our findings. Moreover, for particularly complex cases of BLDH, a single surgical approach may not provide adequate decompression in all scenarios (22). Future research directions could include: (1) investigating combined surgical approaches (TFA with ILA), or (2) incorporating advanced biomaterials to potentially reduce recurrence risks and improve clinical outcomes.

## 6 Conclusion

This study suggests that, under the same standardized postoperative mecobalamin treatment background, both TFA and ILA are effective for the treatment of BLDH. Although overall clinical efficacy and complication rates were comparable between the two groups, ILA was associated with better operative efficiency and superior early postoperative recovery, as reflected by shorter operative time, fewer fluoroscopy exposures, and better short-term improvements in VAS and ODI scores. The observed differences should be interpreted as reflecting the comparative performance of the two surgical approaches rather than the independent or synergistic effect of mecobalamin.

## Data Availability

The original contributions presented in the study are included in the article/supplementary material, further inquiries can be directed to the corresponding author.
